# Supplementary material for: Plant DNA metabarcoding of lake sediments: How does it represent the contemporary vegetation
Source: PLoS One. 2018 Apr 17;13(4):e0195403. doi: 10.1371/journal.pone.0195403 (PMC5903670; doi:10.1371/journal.pone.0195403)
Supplement: S1 Appendix — (DOCX) [file pone.0195403.s001.docx]

**S1 Appendix. Comments on true and false positives**

No combination of filtering criteria allowed us to discard only false positives, possibly because different sources of error give different patterns of sequence reads [1, 2]. For example, *Pinus* was lost in filtering due to its occurrence in two negative controls. While it has been reported as a laboratory contaminant elsewhere [3], we recently found an excellent match between *Pinus* DNA and historical vegetation changes [4]. In this current study, all records of *Pinus* are likely true positives. Its occurrence in negative controls was probably caused by drop contamination, as during PCR the FP samples were next to samples from lakes surrounded by pine forest. In contrast, PCR or sequencing errors [5] likely caused DNA of *Andromeda polyfolia*, recorded in the same samples, to be wrongly assigned to the rare species *Chamaedaphne calyculata,* as these species differ in only one base pair (8 or 9 T at poly-T region). The large number of true positives lost during filtering (see yellow cells in Table S1) shows there is considerable potential to increase the information gained from eDNA. This may be achieved by improved technical and bioinformatic methods and by modelling [6-9]. In particular, fitting statistical models in which the FP and TP rates vary as a function of species and lake traits may help identify the factors leading to true and false positives.

In total, 220 sequences were filtered out (Table S4). Twenty-three sequences of 17 families were only found in negative controls (Table S4), and may represent contamination of the reagents [5]. This type of false positives generally pose no problem to interpretation of metabarcoding data as they are easy to filter out. A further 52 sequences were more common in negative controls than in samples (Table S4). Some of these are likely to represent true positives e.g. *Bistorta vivipara, Cirsium* sp*, Viola biflora,* and *Ranunculus*, which due to e.g. drop contamination or aerosols end up in the negative controls where they may be amplified to high numbers since there is low template concentration. Others may represent contamination of reagents, e.g. *Glycine soja* (soybean). A further 29 sequences were more frequent in lake than control samples and 116 were only found in lake samples, in total 66% of the sequences (Table S4). This includes e.g. Anacardiaceae (*Anacardium occidentale,* cashew)*,* which we found with 263 reads in one PCR repeats from Paulan Jávri. This taxa has also been found in high altitude lake in Pamir [10] and East Africa [11] and high latitude lake in NW Russia [12], suggesting that food contamination is a possibility. However, we think contamination of reagents is not a likely explanation for the majority of these records. For example, *Brunfelsia densifolia* (Serpentine Hill rain tree), *Bagassa guianensis, Guarea silvatica* (both some use as timber), *Goupia glabra* (tropical, no common use found online), *Abarema jupunba* (a plant with some medical use), or *Philenoptera cyanescens* (used as blue dye) are less likely to be found as contamination in northern laboratories or laboratories were the reagents are produces. More likely, PCR and sequencing artefacts may cause minor changes to the sequence. Especially long mononucleotide repeats of “A” are common in the P6 loop, which may cause problems for the *Taq* polymerase. The majority of these taxa were filtered out due to occurrence in only on PCR repeat, which also suggest that they are artefacts. Many of them are short and when we BLAST them, we find 100% match to more than one taxa. The generally poor quality of DNA in environmental samples may cause a high error rate. This combined with the short length of the P6 loop and the mononucleotide regions, may cause random match to taxa in the reference library. The majority of sequences in Genebank are cultivated plants and therefor random match may erroneous be interpreted as food contamination. Using a reference library appropriate for the study site (taking into account that past taxa may differ from current ones on the case of aDNA analyses), may avoid the majority of these false positives. Indeed, Lamb et al. [13] have shown that using a reference library restricted to taxa growing at the study site improved the match between root fragments identified and aboveground richness. In addition, using a local reference library may allow a lower cut off level to be used and thereby reducing the loss of true positives.

**References**

1. Lusk RW. Diverse and Widespread Contamination Evident in the Unmapped Depths of High Throughput Sequencing Data. PLoS ONE. 2014;9(10):e110808. doi: 10.1371/journal.pone.0110808. PubMed PMID: PMC4213012.

2. Porter TM, Golding GB, King C, Froese D, Zazula G, Poinar HN. Amplicon pyrosequencing late Pleistocene permafrost: the removal of putative contaminant sequences and small-scale reproducibility. Mol Ecol Res. 2013;13(5):798-810. doi: 10.1111/1755-0998.12124.

3. Murray DC, Pearson SG, Fullagar R, Chase BM, Houston J, Atchison J, et al. High-throughput sequencing of ancient plant and mammal DNA preserved in herbivore middens. Quat Sci Rev. 2012;58:135-45. doi: <http://dx.doi.org/10.1016/j.quascirev.2012.10.021>.

4. Sjögren P, Edwards ME, Gielly L, Langdon C, Croudace IW, Merkel MKF, et al. Lake sedimentary DNA accurately records 20th century introductions of exotic conifers in Scotland. New Phytol. 2017;213:929-41. doi: 10.1111/nph.14199.

5. Thomsen PF, Willerslev E. Environmental DNA - An emerging tool in conservation for monitoring past and present biodiversity. Biol Conserv. 2015;183:4-18. doi: 10.1016/j.biocon.2014.11.019.

6. Parducci L, Bennett KD, Ficetola GF, Alsos IG, Suyama Y, Wood JR, et al. Transley Reviews: Ancient plant DNA from lake sediments. New Phytol. 2017;214(3):924-42. doi: 10.1111/nph.14470.

7. Schmidt BR, Kéry M, Ursenbacher S, Hyman OJ, Collins JP. Site occupancy models in the analysis of environmental DNA presence/absence surveys: a case study of an emerging amphibian pathogen. Methods in Ecology and Evolution. 2013;4(7):646-53. doi: 10.1111/2041-210X.12052.

8. Coissac E, Hollingsworth PM, Lavergne S, Taberlet P. From barcodes to genomes: extending the concept of DNA barcoding. Mol Ecol. 2016:1423-8. doi: 10.1111/mec.13549.

9. Pedersen MW, Ruter A, Schweger C, Friebe H, Staff RA, Kjeldsen KK, et al. Postglacial viability and colonization in North America’s ice-free corridor. Nature. 2016;537:45-9. doi: 10.1038/nature19085

<http://www.nature.com/nature/journal/vaop/ncurrent/abs/nature19085.html#supplementary-information>.

10. Heinecke L, Epp LS, Reschke M, Stoof-Leichsenring KR, Mischke S, Plessen B, et al. Aquatic macrophyte dynamics in Lake Karakul (Eastern Pamir) over the last 29 cal ka revealed by sedimentary ancient DNA and geochemical analyses of macrofossil remains. J Paleolimnol. 2017. doi: 10.1007/s10933-017-9986-7.

11. Boessenkool S, McGlynn G, Epp LS, Taylor D, Pimentel M, Gizaw A, et al. Use of ancient sedimentary DNA as a novel conservation tool for high-altitude tropical biodiversity. Conserv Biol. 2014;28(2):446-55. doi: 10.1111/cobi.12195.

12. Parducci L, Väliranta M, Salonen JS, Ronkainen T, Matetovici I, Fontana SL, et al. Proxy comparison in ancient peat sediments: pollen, macrofossil and plant DNA. Philos Trans R Soc London Ser B. 2015;370(1660):20130382. doi: 10.1098/rstb.2013.0382.

13. Lamb EG, Winsley T, Piper CL, Freidrich SA, Siciliano SD. A high-throughput belowground plant diversity assay using next-generation sequencing of the trnL intron. Plant and Soil. 2016;404(1):361-72. doi: 10.1007/s11104-016-2852-y.
