# Supplementary material for: Plant DNA metabarcoding of lake sediments: How does it represent the contemporary vegetation
Source: PLoS One. 2018 Apr 17;13(4):e0195403. doi: 10.1371/journal.pone.0195403 (PMC5903670; doi:10.1371/journal.pone.0195403)
Supplement: S1 Table — (<2 m and/or larger surveys) at 11 lakes in northern Norway. Number refers to the highest abundance recorded among 2–17 vegetation polygons in the larger vegetation surveys (1 = rare, 2 = scattered, 3 = frequent, and 4 = dominant). Thus, 2316 records were combined to give one vegetation record per species and lake, in total 1000 records. Taxa match represent taxa that could potentially be identified by the molecular method used: ND = no data in reference library, ID incomp = could not be identified in DNA because the vegetation is incomplete identified, <12 bp = filtered out in initial filtering steps due to short sequence length. Max = the maximum abundance score observed at any of the lakes. The lakes names are A-tjern (A-tj), Brennskogtjørna (Bren), Einletvatnet (Einl), Finnvatnet (Finn), Gauptjern (Gaup), Jula Jávri (Jula), Lakselvhøgda (Laks), Lauvås (Lauv), Øvre Æråsvatnet (Ovre), Paulan Jávri (Paul), and Rottjern (Rott). See colour codes below. Hatched colour refer to DNA-vegetation match at higher taxonomic level (e.g. Salix). (DOCX) [file pone.0195403.s002.docx]

**S1 Table. All taxa recorded in the vegetation surveys** (<2 m and/or larger surveys) at 11 lakes in northern Norway. Number refers to the highest abundance recorded among 2-17 vegetation polygons in the larger vegetation surveys (1=rare, 2=scattered, 3=frequent, and 4=dominant). Thus, 2316 records were combined to give one vegetation record per species and lake, in total 1000 records. Taxa match represent taxa that could potentially be identified by the molecular method used: ND=no data in reference library, ID incomp=could not be identified in DNA because the vegetation is incomplete identified, <12 bp= filtered out in initial filtering steps due to short sequence length. Max=the maximum abundance score observed at any of the lakes. The lakes names are A-tjern (A-tj), Brennskogtjørna (Bren), Einletvatnet (Einl), Finnvatnet (Finn), Gauptjern (Gaup), Jula Jávri (Jula), Lakselvhøgda (Laks), Lauvås (Lauv), Øvre Æråsvatnet (Ovre), Paulan Jávri (Paul), and Rottjern (Rott). See colour codes below. Hatched colour refer to DNA-vegetation match at higher taxonomic level (e.g. *Salix*).

| **Family** | **Taxa** | **Taxa match** | **A-tj** | **Bren** | **Einl** | | **Finn** | **Gaup** | **Jula** | **Laks** | **Lauv** | **Ovre** | **Paul** | **Rott** | **Max** | |  |
| --- | --- | --- | --- | --- | --- | --- | --- | --- | --- | --- | --- | --- | --- | --- | --- | --- | --- |
| Alliaceae | *Allium schoenoprasum* | All_sch |  |  |  | |  |  |  |  | 1 |  |  |  | 1 | |  |
| Apiaceae | *Angelica archangelica* | Ang_arc |  | 2 |  | |  | 2 |  |  |  |  | 1 | 1 | 2 | |  |
| Apiaceae | *Angelica sylvestris* | Ang_syl | 1 |  | 2 | | 1 | 1 |  |  | 1 | 1 |  |  | 2 | |  |
| Apiaceae | *Anthriscus sylvestris* | Ant_syl |  |  |  | |  | 2 |  |  |  |  |  | 1 | 2 | |  |
| Asteraceae | *Achillea millefolium* | Ach_mil |  |  | 2 | |  | 2 |  |  |  | 1 |  |  | 2 | |  |
| Asteraceae | *Antennaria alpina* | Ant_sp |  |  |  | |  |  | 1 |  |  |  | 1 |  | 1 | |  |
| Asteraceae | *Antennaria dioica* | Ant_sp |  |  |  | |  |  |  |  |  | 1 | 2 |  | 2 | |  |
| Asteraceae | *Cicerbita alpina* | ND | 2 |  | 3 | |  |  |  | 1 |  | 2 |  |  | 3 | |  |
| Asteraceae | *Cirsium heterophyllum* | Cir_het | 3 | 2 |  | |  | 1 |  |  |  | 2 | 1 | 2 | 3 | |  |
| Asteraceae | *Crepis paludosa* | Cre_pal |  |  |  | |  |  |  |  |  | 1 |  |  | 1 | |  |
| Asteraceae | *Erigeron uniflorus* | Eri_uni |  |  |  | |  |  | 1 |  |  |  |  |  | 1 | |  |
| Asteraceae | *Hieracium alpinum* | Hier_alp |  |  | 1 | |  |  | 1 | 1 |  | 1 | 1 |  | 1 | |  |
| Asteraceae | *Hieracium* sect. *Hieracium* | ID incomp |  |  |  | |  |  |  |  |  | 1 |  |  | 1 | |  |
| Asteraceae | *Hieracium* sp*.* | ID incomp | 1 | 1 |  | |  | 1 |  | 1 |  | 2 | 1 | 1 | 2 | |  |
| Asteraceae | *Omalotheca norvegica* | Oma_nor |  |  | 1 | |  |  |  |  |  | 1 |  |  | 1 | |  |
| Asteraceae | *Omalotheca supina* | Oma_sup |  |  |  | |  |  |  |  |  |  | 1 |  | 1 | |  |
| Asteraceae | *Petasites frigidus* | Pet_fri |  |  |  | |  |  | 1 |  |  |  |  |  | 1 | |  |
| Asteraceae | *Saussurea alpina* | Sau_alp | 2 | 3 |  | | 2 | 3 | 1 |  | 1 | 1 | 1 | 2 | 3 | |  |
| Asteraceae | *Scorzoneroides autumnalis ssp. borealis* | ND |  |  |  | | 1 | 1 | 1 |  | 1 |  |  |  | 1 | |  |
| Asteraceae | *Solidago virgaurea* | Sol_vir | 1 | 3 | 3 | |  | 3 | 1 | 1 | 1 | 2 | 1 | 3 | 3 | |  |
| Asteraceae | *Taraxacum* sp. | Tar_sp |  |  | 2 | | 1 |  | 1 |  |  | 1 | 2 |  | 2 | |  |
| Asteraceae | *Tussilago farfara* | Tus_far |  | 1 |  | |  |  |  |  |  |  |  |  | 1 | |  |
| Betulaceae | *Alnus incana* | Aln_inc | 3 | 3 |  | |  | 2 |  |  |  | 1 |  | 3 | 3 | |  |
| Betulaceae | *Betula nana* | Bet_sp |  |  | 3 | | 3 | 2 | 3 | 2 | 3 | 3 | 4 | 1 | 4 | |  |
| Betulaceae | *Betula pubescens subsp. tortuosa* | Bet_sp | 4 | 4 | 4 | | 4 | 4 |  | 4 | 4 | 4 | 1 | 4 | 4 | |  |
| Boraginaceae | *Myosotis arvensis* | Myo_arv |  | 1 |  | |  | 1 |  |  |  |  |  | 1 | 1 | |  |
| Brassicaceae | *Arabis alpina* | Ara_alp |  |  |  | |  |  | 1 |  |  |  | 1 |  | 1 | |  |
| Brassicaceae | *Cardamine pratense* | Car_pra |  |  |  | |  |  |  |  |  |  |  | 1 | 1 | |  |
| Brassicaceae | *Draba nivalis* | Dra_niv |  |  |  | |  |  | 1 |  |  |  |  |  | 1 | |  |
| Brassicaceae | *Subularia aquatica* | Sub_aqu |  |  | 1 | |  |  |  |  |  | 3 |  |  | 3 | |  |
| Campanulaceae | *Campanula rotundifolia* | Cam_rot | 1 | 1 |  | | 1 |  | 2 | 1 | 2 | 1 | 2 |  | 2 | |  |
| Caryophyllaceae | *Cerastium alpinum ssp. alpinum* | Cer_alp |  |  |  | |  |  |  |  |  |  | 1 |  | 1 | |  |
| Caryophyllaceae | *Cerastium fontanum* | Cer_fon |  | 1 |  | |  |  |  |  |  | 1 |  |  | 1 | |  |
| Caryophyllaceae | *Lychnis flos-cuculi* | Lyc_flo |  |  |  | |  |  |  |  |  | 1 |  |  | 1 | |  |
| Caryophyllaceae | *Silene acaulis* | Sil_aca |  | 1 |  | |  |  | 2 |  |  |  | 1 |  | 2 | |  |
| Caryophyllaceae | *Stellaria nemorum* | Ste_nem |  |  |  | |  |  |  |  |  | 1 |  | 1 | 1 | |  |
| Cornaceae | *Chamaepericlymenum suecicum* | Cha_sue | 3 |  | 3 | | 2 | 3 |  | 4 | 3 | 4 | 2 | 4 | 4 | |  |
| Crassulaceae | *Rhodiola rosea* | Rho_ros |  |  |  | |  |  | 1 |  |  | 1 |  |  | 1 | |  |
| Crassulaceae | *Sedum rosea* | Sed_ros |  |  |  | |  |  | 1 |  |  |  |  |  | 1 | |  |
| Cupressaceae | *Juniperus communis* | Jun_com | 3 | 2 |  | | 1 | 2 |  | 2 | 1 | 1 | 3 | 3 | 3 | |  |
| Cyperaceae | *Carex adelostoma* | Car_sp |  |  |  | |  |  |  |  | 1 |  | 3 |  | 3 | |  |
| Cyperaceae | *Carex aquatilis* | Car_sp |  |  | 4 | | 4 |  |  |  |  | 1 |  |  | 4 | |  |
| Cyperaceae | *Carex bigelowii* | Car_sp |  |  | 1 | | 1 |  | 4 | 3 |  |  | 1 |  | 4 | |  |
| Cyperaceae | *Carex brunnescens* | Car_sp |  |  | 4 | | 1 | 1 |  |  |  |  |  |  | 4 | |  |
| Cyperaceae | *Carex canescens* | Car_sp |  | 1 | 2 | |  |  |  |  | 1 | 1 |  | 2 | 2 | |  |
| Cyperaceae | *Carex* cf. *adelostoma* | Car_sp |  |  |  | |  |  |  |  |  |  |  | 1 | 1 | |  |
| Cyperaceae | *Carex* cf*. brunnescens* | Car_sp |  |  |  | |  |  |  |  |  |  |  | 2 | 2 | |  |
| Cyperaceae | *Carex* cf*. capillaris* ssp. *capillaris* | Car_sp |  | 2 | 1 | |  |  |  |  |  |  | 1 |  | 2 | |  |
| Cyperaceae | *Carex concolor (*Syn. *C. aquatilis* ssp*. stans)* | Car_sp |  |  |  | |  |  | 3 |  |  |  |  |  | 3 | |  |
| Cyperaceae | *Carex diandra* | Car_dia |  |  |  | |  |  |  |  |  |  |  | 2 | 2 | |  |
| Cyperaceae | *Carex dioica* | Car_sp |  | 1 |  | |  | 2 |  |  |  |  |  |  | 2 | |  |
| Cyperaceae | *Carex echinata* | Car_sp |  |  |  | | 2 |  |  | 4 | 1 | 2 |  |  | 4 | |  |
| Cyperaceae | *Carex flava* | Car_sp | 3 | 2 |  | | 2 |  |  |  | 2 |  |  |  | 3 | |  |
| Cyperaceae | *Carex lachenalii* | Car_lac |  |  |  | |  |  |  |  |  | 2 | 1 |  | 2 | |  |
| Cyperaceae | *Carex lasiocarpa* | Car_las | 3 |  |  | |  | 3 |  |  |  |  |  | 4 | 4 | |  |
| Cyperaceae | *Carex nigra* ssp. *juncella* | Car_sp | 3 |  | 3 | | 2 |  | 2 |  | 1 | 2 | 1 |  | 3 | |  |
| Cyperaceae | *Carex norvegica* | Car_sp |  | 2 |  | |  |  | 3 |  |  |  | 1 |  | 3 | |  |
| Cyperaceae | *Carex paleacea hybrid* | Car_sp |  |  |  | |  |  |  |  | 1 |  |  |  | 1 | |  |
| Cyperaceae | *Carex panicea* | Car_pan |  |  |  | | 2 |  |  |  | 3 |  |  |  | 3 | |  |
| Cyperaceae | *Carex pauciflora* | Car_pac |  |  |  | |  |  |  |  |  | 3 |  |  | 3 | |  |
| Cyperaceae | *Carex paupercula* | Car_paup |  |  |  | | 2 | 1 |  | 3 | 1 | 2 |  |  | 3 | |  |
| Cyperaceae | *Carex rariflora* | Car_sp | 2 |  | 2 | | 1 |  |  |  | 1 | 2 |  |  | 2 | |  |
| Cyperaceae | *Carex rostrata* | Car_sp | 3 | 2 | 4 | | 4 | 3 |  | 1 | 1 | 2 |  | 4 | 4 | |  |
| Cyperaceae | *Carex saxatilis* | Car_sp | 1 |  | 1 | |  |  | 3 |  |  |  | 4 |  | 4 | |  |
| Cyperaceae | *Carex* sp. | Car_sp |  |  | 4 | | 2 | 1 | 4 | 1 | 1 | 2 | 4 | 1 | 4 | |  |
| Cyperaceae | *Carex vacillans* | Car_sp |  |  |  | |  |  |  |  | 1 |  |  |  | 1 | |  |
| Cyperaceae | *Carex vaginata* | Car_vag | 2 | 3 |  | | 2 |  |  | 1 | 1 |  | 1 |  | 3 | |  |
| Cyperaceae | *Eriophorum angustifolium* | Eri_sp |  | 2 | 4 | | 3 | 2 | 4 | 3 | 2 | 3 | 3 | 2 | 4 | |  |
| Cyperaceae | *Eriophorum scheuchzerii* | Eri_sch |  |  |  | |  |  | 1 | 1 |  |  | 1 |  | 1 | |  |
| Cyperaceae | *Eriophorum* sp*.* | ID incomp |  | 1 |  | |  |  |  |  |  |  |  |  | 1 | |  |
| Cyperaceae | *Eriophorum vaginatum* | Eri_sp | 2 |  | 4 | | 3 | 1 |  | 1 | 4 | 3 |  | 1 | 4 | |  |
| Cyperaceae | *Trichophorum alpinum* | Tri_alp | 3 |  |  | |  |  |  | 1 |  |  |  |  | 3 | |  |
| Cyperaceae | *Trichophorum cespitosum* | Tri_ces |  |  | 3 | | 1 |  |  | 1 | 1 | 3 |  |  | 3 | |  |
| Diapensiaceae | *Diapensia lapponica* | Dia_lap |  |  |  | |  |  |  | 1 |  |  |  |  | 1 | |  |
| Droseraceae | *Drosera longifolia* | Dro_lon |  |  | 1 | | 1 |  |  | 1 | 1 | 1 |  |  | 1 | |  |
| Droseraceae | *Drosera rotundifolia* | Dro_rot | 3 |  | 2 | | 1 |  |  | 1 | 1 | 1 |  |  | 3 | |  |
| Dryopteridaceae | *Dryopteris expansa* | Dry_sp | 3 |  | 4 | | 2 |  |  | 2 | 3 | 3 |  | 2 | 4 | |  |
| Dryopteridaceae | *Dryopteris filix-mas* | Dry_sp |  |  |  | |  |  |  |  |  | 2 |  |  | 2 | |  |
| Dryopteridaceae | *Dryopteris* sp. | Dry_sp |  | 2 |  | |  |  |  |  |  |  |  |  | 2 | |  |
| Equisetaceae | *Equisetum arvense* | <12 bp |  |  | 2 | |  |  | 2 |  |  |  | 1 | 2 | 2 | |  |
| Equisetaceae | *Equisetum fluviatile* | <12 bp |  |  | 2 | |  |  |  |  |  | 2 |  | 4 | 4 | |  |
| Equisetaceae | *Equisetum palustre* | <12 bp | 2 | 2 | 2 | | 1 | 3 | 1 |  |  |  | 1 |  | 3 | |  |
| Equisetaceae | *Equisetum pratense* | <12 bp | 2 |  | 2 | |  | 3 |  |  |  |  |  |  | 3 | |  |
| Equisetaceae | *Equisetum scipoides* | <12 bp |  |  |  | |  | 1 | 1 |  |  |  |  |  | 1 | |  |
| Equisetaceae | *Equisetum* sp. | <12 bp |  |  |  | |  |  |  |  |  |  | 2 |  | 2 | |  |
| Equisetaceae | *Equisetum sylvaticum* | <12 bp | 3 |  | 2 | | 3 |  |  |  | 2 | 3 |  | 3 | 3 | |  |
| Equisetaceae | *Equisetum variegatum* | <12 bp |  | 2 | 2 | | 1 |  | 1 |  |  |  |  |  | 2 | |  |
| Ericaceae | *Andromeda polifolia* | And_pol | 3 | 2 | 3 | | 3 | 2 |  | 2 | 3 | 4 | 1 | 1 | 4 | |  |
| Ericaceae | *Arctostaphylos uva-ursi* | Arc_uva |  | 3 |  | |  |  |  |  |  |  |  |  | 3 | |  |
| Ericaceae | *Arctous alpinus* | Arc_alp |  | 2 | 2 | |  |  | 3 | 2 | 2 | 1 | 2 |  | 3 | |  |
| Ericaceae | *Calluna vulgaris* | Cal_vul |  |  | 3 | | 2 |  |  | 4 | 2 | 4 |  |  | 4 | |  |
| Ericaceae | *Cassiope tetragona* | Cas_tet |  | 1 |  | |  |  | 3 |  |  | 1 | 1 |  | 3 | |  |
| Ericaceae | *Empetrum nigrum* | Emp_nig | 4 | 4 | 4 | | 3 | 4 | 4 | 4 | 4 | 4 | 4 | 4 | 4 | |  |
| Ericaceae | *Harrimanella hypnoides* | ND |  | 1 |  | |  |  | 1 |  |  |  | 1 |  | 1 | |  |
| Ericaceae | *Loiseleuria procumbens* | Loi_pro |  |  | 2 | |  |  | 2 | 2 | 2 |  | 2 |  | 2 | |  |
| Ericaceae | *Oxycoccus microcarpus* | Oxy_mic | 3 | 1 | 2 | | 1 | 2 |  |  | 1 |  |  | 2 | 3 | |  |
| Ericaceae | *Phyllodoce caeruela* | Phy_cae | 4 | 2 |  | |  | 2 | 1 |  |  |  | 1 | 1 | 4 | |  |
| Ericaceae | *Rhododendron lapponicum* | Rho_lap |  | 1 |  | |  |  |  |  |  |  |  |  | 1 | |  |
| Ericaceae | *Vaccinium myrtillus* | Vac_sp | 4 | 3 | 3 | | 3 | 3 |  | 3 | 2 | 3 | 3 | 4 | 4 | |  |
| Ericaceae | *Vaccinium uliginosum* | Vac_uli | 4 | 1 | 3 | | 2 | 3 | 2 | 2 | 3 | 4 | 3 | 3 | 4 | |  |
| Ericaceae | *Vaccinium vitis-idaea* | Vac_sp | 4 | 1 | 3 | | 1 | 4 | 2 | 2 | 3 | 2 | 2 | 4 | 4 | |  |
| Fabaceae | *Astragalus alpinus* | Ast_sp |  |  |  | |  |  | 1 |  |  |  | 1 |  | 1 | |  |
| Fabaceae | *Astragalus alpinus* ssp. *arcticus* | Ast_sp |  | 1 |  | |  |  |  |  |  |  |  |  | 1 | |  |
| Fabaceae | *Astragalus frigidus* | Ast_sp |  |  |  | |  |  |  |  |  |  | 1 |  | 1 | |  |
| Fabaceae | *Lathyrus pratensis* | Lat_pra |  |  |  | |  |  |  |  |  | 2 |  |  | 2 | |  |
| Fabaceae | *Lotus corniculatus* | Lot_cor |  |  | 1 | |  |  |  |  |  | 1 |  |  | 1 | |  |
| Fabaceae | *Trifolium pratense* | Tri_pra |  |  |  | |  | 2 |  |  |  |  |  |  | 2 | |  |
| Fabaceae | *Trifolium repens* | Tri_rep |  | 1 |  | |  | 2 |  |  | 1 |  |  |  | 2 | |  |
| Fabaceae | *Vicci cracca* | Vic_cra |  |  |  | |  |  |  |  | 1 | 2 |  |  | 2 | |  |
| Gentianaceae | *Comastoma tenellum* | Com_ten |  |  |  | |  |  | 1 |  |  |  |  |  | 1 | |  |
| Gentianaceae | *Gentiana nivalis* | Gen_niv |  |  |  | |  |  |  |  |  |  | 1 |  | 1 | |  |
| Geraniaceae | *Geranium sylvaticum* | Ger_syl | 2 | 2 | 2 | |  | 2 |  | 2 | 1 | 3 | 3 | 3 | 3 | |  |
| Grossulariaceae | *Ribes* cf*. spicatum* | Rib_spi |  |  |  | |  |  |  |  |  |  |  | 2 | 2 | |  |
| Haloragaceae | *Myriophyllum alterniflorum* | Myr_alt | 3 |  | 4 | |  |  |  |  | 4 | 4 |  |  | 4 | |  |
| Isoetes lacustris | *Isoetes lacustris* | Iso_sp |  |  | 1 | |  |  |  |  |  | 1 |  |  | 1 | |  |
| Juncaceae | *Juncus alpinoarcticulatus* | Jun_alp |  | 1 |  | |  |  |  |  |  |  |  |  | 1 | |  |
| Juncaceae | *Juncus balticus* | Jun_sp |  |  |  | |  |  |  |  | 4 |  |  |  | 4 | |  |
| Juncaceae | *Juncus filiformis* | Jun_sp |  |  | 2 | | 1 |  |  |  | 3 | 2 | 1 |  | 3 | |  |
| Juncaceae | *Juncus trifidus* | Jun_trif |  |  |  | |  |  |  | 4 | 2 | 2 | 3 |  | 4 | |  |
| Juncaceae | *Juncus triglumis* | Jun_trig |  | 1 |  | |  |  |  |  |  |  | 1 |  | 1 | |  |
| Juncaceae | *Luzula mulitflora* | Luz_sp | 2 |  | 2 | |  |  | 1 |  | 1 | 2 | 1 |  | 2 | |  |
| Juncaceae | *Luzula pilosa* | Luz_pil | 1 | 3 |  | |  | 1 |  |  |  | 2 |  | 2 | 3 | |  |
| Juncaceae | *Luzula spicata* | Luz_sp |  |  |  | |  |  | 1 |  |  |  | 1 |  | 1 | |  |
| Juncaceae | *Luzula sudetica* | Luz_sp |  |  |  | | 1 |  |  |  | 1 | 1 | 1 |  | 1 | |  |
| Juncaginaceae | *Triglochin palustris* | Tri_pal |  |  | 2 | | 1 |  |  |  | 1 |  |  |  | 2 | |  |
| Lentibulariaceae | *Pinguicula alpina* | Ping_sp |  |  |  | |  |  | 1 |  |  |  | 1 |  | 1 | |  |
| Lentibulariaceae | *Pinguicula vulgaris* | Ping_sp | 3 | 3 | 1 | | 2 | 3 |  | 2 | 1 | 1 |  | 1 | 3 | |  |
| Lentibulariaceae | *Utricularia minor* | Utr_min | 1 |  |  | |  |  |  |  |  |  |  | 1 | 1 | |  |
| Linnaeaceae | *Linnaea borealis* | Lin_bor | 1 | 3 |  | |  | 3 |  |  |  | 1 |  | 3 | 3 | |  |
| Lycopodiaceae | *Diphasiastrum alpinum* | Lycopod |  |  |  | |  |  | 1 | 1 |  |  | 2 |  | 2 | |  |
| Lycopodiaceae | *Diphasiastrum complanatum* ssp. *montellii* | Lycopod |  | 2 |  | |  |  |  |  |  |  |  |  | 2 | |  |
| Lycopodiaceae | *Huperzia selago* | Hyp_sel | 2 | 1 | 1 | |  |  | 1 | 1 | 1 | 1 | 1 | 2 | 2 | |  |
| Lycopodiaceae | *Lycopodium annotinum ssp. annotinum* | Lycopod | 3 | 3 | 1 | |  | 3 | 1 | 1 |  |  | 1 | 3 | 3 | |  |
| Lycopodiaceae | *Lycopodium clavatum ssp. monostachyon* | Lycopod | 1 |  | 3 | |  |  |  | 1 | 1 |  | 1 | 1 | 3 | |  |
| Melanthiaceae | *Paris quadrifolia* | Par_qua |  |  |  | |  |  |  |  |  |  |  | 2 | 2 | |  |
| Menyanthaceae | *Menyanthes trifoliata* | Men_tri | 4 |  | 4 | | 3 | 3 |  | 3 | 4 | 1 |  | 4 | 4 | |  |
| Montiaceae | *Montia fontana* | Mon_fon |  |  |  | |  |  |  |  | 1 |  |  |  | 1 | |  |
| Nartheciaceae | *Narthecium ossifragum* | Nar_oss |  |  | 2 | |  |  |  |  |  |  |  |  | 2 | |  |
| Nymphaeaceae | *Nuphar pumila* | Nup_pum |  |  |  | |  |  |  |  |  |  |  | 3 | 3 | |  |
| Onagraceae | *Chamerion angustifolium* | Cha_ang | 2 | 3 | 3 | |  | 3 |  |  |  |  |  | 2 | 3 | |  |
| Onagraceae | *Epilobium anagallidifolium* | Epi_ana |  |  |  | |  |  | 1 |  |  |  | 1 |  | 1 | |  |
| Onagraceae | *Epilobium palustre* | Epi_pal |  |  |  | |  |  |  |  | 1 |  |  | 1 | 1 | |  |
| Orchidaceae | *Coeloglossum viride* | Dac_sp |  | 1 |  | |  |  |  |  |  |  | 1 |  | 1 | |  |
| Orchidaceae | *Dactylorhiza* cf*. fuchsii* | Dac_sp |  | 1 | 1 | |  |  |  |  |  | 1 |  | 1 | 1 | |  |
| Orchidaceae | *Dactylorhiza maculata* | Dac_sp | 2 |  | 1 | | 1 | 2 |  | 1 | 1 | 1 |  |  | 2 | |  |
| Orchidaceae | *Epipactis atrorubens* | Epi_atr |  |  |  | |  |  |  |  |  |  |  | 1 | 1 | |  |
| Orchidaceae | *Goodera repens* | Goo_rep |  | 1 |  | |  |  |  |  |  |  |  |  | 1 | |  |
| Orchidaceae | *Listera cordata* | Lis_cor |  | 1 |  | |  | 1 |  |  |  |  |  |  | 1 | |  |
| Orobanchaceae | *Bartsia alpina* | Bar_alp |  | 3 | 1 | | 2 | 2 | 1 | 1 |  |  | 1 | 1 | 3 | |  |
| Orobanchaceae | *Euphrasia hyperborea* | Eup_sp |  |  |  | |  |  |  | 1 |  |  |  |  | 1 | |  |
| Orobanchaceae | *Euphrasia stricta* | Eup_sp |  |  |  | |  | 2 |  |  |  |  |  |  | 2 | |  |
| Orobanchaceae | *Euphrasia wettsteinii* | Eup_sp |  | 2 |  | | 1 |  | 1 |  |  | 1 |  |  | 2 | |  |
| Orobanchaceae | *Euphrasis* sp*.* | Eup_sp | 1 |  |  | |  |  |  |  |  |  |  |  | 1 | |  |
| Orobanchaceae | *Melampyrum pratense* | Mel_pra | 3 | 2 |  | |  | 3 |  |  |  | 3 |  | 3 | 3 | |  |
| Orobanchaceae | *Melampyrum sylvaticum* | Mel_syl | 2 | 2 |  | |  | 3 |  | 1 | 1 | 3 | 1 | 3 | 3 | |  |
| Orobanchaceae | *Pedicularis* cf. *lapponica* | Ped_lap |  | 1 |  | |  | 1 | 1 |  |  |  | 1 |  | 1 | |  |
| Orobanchaceae | *Pedicularis hirsuta* | Ped_hir |  |  |  | |  |  | 1 |  |  |  |  |  | 1 | |  |
| Orobanchaceae | *Pedicularis palustris* | Ped_pal |  |  | 2 | | 1 |  |  |  |  | 1 |  |  | 2 | |  |
| Orobanchaceae | *Rhinanthus minor* | Rhi_min |  | 2 |  | | 1 | 2 |  |  |  | 1 | 1 |  | 2 | |  |
| Parnassiaceae | *Parnassia palustris* | Par_pal | 3 | 3 |  | |  | 2 |  |  | 2 | 1 | 1 | 2 | 3 | |  |
| Pinaceae | *Picea abies* | Pic_abi | 2 |  |  | |  |  |  |  |  | 2 |  |  | 2 | |  |
| Pinaceae | *Pinus sylvestris* | Pin_syl | 4 | 4 |  | |  | 3 |  |  |  | 2 | 1 | 1 | 4 | |  |
| Plantaginaceae | *Callitriche palustris* | Calli_pal |  |  | 1 | |  |  |  |  |  |  |  |  | 1 | |  |
| Plantaginaceae | *Hippuris vulgare* | Hip_vul |  |  | 2 |  | |  |  |  | 2 | 2 |  | 2 | | 2 |  |
| Plantaginaceae | *Veronica alpina ssp. alpina* | Ver_alp |  |  |  | |  |  | 1 |  |  |  | 1 |  | 1 | |  |
| Poaceae | *Agrostis capillaris* | Poaceae2 | 1 |  |  | | 2 | 1 |  |  | 2 | 2 |  |  | 2 | |  |
| Poaceae | *Agrostis mertensii* | Agr_mer |  |  |  | |  |  | 3 |  |  |  | 1 |  | 3 | |  |
| Poaceae | *Agrostis* sp*.* | ID incomp | 1 |  |  | |  |  |  |  |  |  |  | 2 | 2 | |  |
| Poaceae | *Anthoxanthum nipponicum* | Ant_nip |  |  | 2 | |  |  | 2 | 1 |  |  | 1 |  | 2 | |  |
| Poaceae | *Avenella flexuosa* | Ave_fle | 3 | 4 | 4 | | 3 | 3 | 1 | 3 | 2 | 2 | 3 | 3 | 4 | |  |
| Poaceae | *Calamagrostis lapponica* ssp. *lapponica* | Poaceae2 | 3 |  |  | |  |  | 1 |  |  |  | 1 |  | 3 | |  |
| Poaceae | *Calamagrostis neglecta* ssp. *groenlandica* | Poaceae |  |  | 3 | |  |  |  |  |  |  | 3 |  | 3 | |  |
| Poaceae | *Calamagrostis neglecta* ssp*. neglecta* | Poaceae2 | 2 | 2 | 2 | | 1 | 2 |  |  |  | 2 | 3 | 2 | 3 | |  |
| Poaceae | *Calamagrostis phragmitoides* | Poaceae2 |  |  | 4 | | 1 |  |  | 1 | 2 | 2 | 1 | 3 | 4 | |  |
| Poaceae | *Deschampsia caespitosa* | Poaceae | 2 | 3 | 3 | | 2 | 2 |  | 1 | 4 | 2 | 1 | 2 | 4 | |  |
| Poaceae | *Deschampsia sp.* | Poaceae |  |  |  | |  |  | 1 |  |  |  |  |  | 1 | |  |
| Poaceae | *Elymus caninus* | Ely_can | 1 |  |  | |  |  |  |  |  |  |  |  | 1 | |  |
| Poaceae | *Festuca ovina* | Fes_sp |  |  |  | |  |  |  |  |  |  | 1 |  | 1 | |  |
| Poaceae | *Festuca rubra* | Fes_sp |  | 2 |  | | 2 | 2 | 1 |  | 2 | 2 | 1 |  | 2 | |  |
| Poaceae | *Festuca vivipara* | ND | 1 |  |  | | 2 |  |  | 1 | 2 | 2 |  |  | 2 | |  |
| Poaceae | *Hierochloe alpina* | Hie_alp |  |  |  | |  |  |  |  |  |  | 1 |  | 1 | |  |
| Poaceae | *Hierochloe odorata ssp. odorata* | Hie_odo |  |  |  | |  |  |  |  |  | 2 |  |  | 2 | |  |
| Poaceae | *Melica nutans* | Mel_nut | 2 | 1 |  | |  |  |  |  |  |  |  | 2 | 2 | |  |
| Poaceae | *Molinia carulea* | Mol_car |  |  |  | |  |  |  |  |  | 2 |  |  | 2 | |  |
| Poaceae | *Nardus stricta* | Nar_str |  |  | 3 | | 4 |  |  | 4 | 2 | 3 | 1 |  | 4 | |  |
| Poaceae | *Phleum alpinum* | Phl_alp |  |  |  | |  | 1 |  |  |  | 1 | 2 |  | 2 | |  |
| Poaceae | *Poa alpina* | Poa_alp |  | 2 |  | |  |  |  |  |  |  |  |  | 2 | |  |
| Poaceae | *Poa alpina* var*. alpina* | Poa_alp |  |  |  | |  |  | 2 |  |  |  | 1 |  | 2 | |  |
| Poaceae | *Poa alpina* var. *vivipara* | Poa_alp |  |  |  | |  |  | 2 |  |  |  | 1 |  | 2 | |  |
| Poaceae | *Poa annua* | Poa_ann |  |  | 4 | |  |  |  |  |  |  |  |  | 4 | |  |
| Poaceae | *Poa nemoralis* | Poa_sp | 1 |  |  | |  |  |  |  |  |  |  |  | 1 | |  |
| Poaceae | *Poa pratensis* | Poa_sp |  |  |  | |  |  |  |  | 1 |  |  |  | 1 | |  |
| Poaceae | Poaceae total | ID incomp |  |  |  | |  |  | 3 |  | 4 | 3 | 4 |  | 4 | |  |
| Poaceae | *Trisetum spicatum* | Tri_spi |  |  |  | |  |  | 2 |  |  |  |  |  | 2 | |  |
| Polygonaceae | *Bistorta vivipara* | Bis_viv | 2 | 3 | 3 | | 1 | 1 | 3 | 1 | 1 | 2 | 2 | 1 | 3 | |  |
| Polygonaceae | *Oxyria digyna* | Oxy_dig |  |  |  | |  |  | 1 |  |  |  | 1 |  | 1 | |  |
| Polygonaceae | *Rumex acetosa* | Rum_sp |  | 1 | 2 | |  |  | 2 |  | 1 | 2 | 2 |  | 2 | |  |
| Polygonaceae | *Rumex acetosella* | Rum_sp |  |  |  | |  |  |  |  |  |  | 1 |  | 1 | |  |
| Polygonaceae | *Rumex longifolius* | Rum_sp |  |  |  | |  |  |  |  |  |  |  |  |  | |  |
| Potamogetonaceae | *Potamogeton alpinus* | Pota_sp |  | 2 | 2 | |  |  |  |  | 2 | 2 |  | 3 | 3 | |  |
| Potamogetonaceae | *Potamogeton gramineus* | Pota_sp | 3 | 3 | 3 | | 2 | 2 |  |  | 2 | 3 |  | 3 | 3 | |  |
| Potamogetonaceae | *Potamogeton praelongus* | Pota_pr |  |  |  | |  |  |  |  |  | 1 |  |  | 1 | |  |
| Potamogetonaceae | *Stuckenia filiformis* | Stuck_sp | 2 | 2 |  | |  | 3 |  |  |  |  |  |  | 3 | |  |
| Primulaceae | *Trientalis europaea* | Tri_eur |  | 2 | 3 | | 1 | 1 |  | 1 | 2 |  |  |  | 3 | |  |
| Pyrolaceae | *Orthilia secunda* | Ort_sec |  | 3 |  | |  | 1 |  |  |  |  |  | 1 | 3 | |  |
| Pyrolaceae | *Pyrola minor* | Pyr_min |  |  |  | |  |  | 1 |  |  | 1 | 1 | 1 | 1 | |  |
| Pyrolaceae | *Pyrola rotundifolia* | Pyr_rot | 1 | 1 |  | |  |  |  |  |  | 1 |  |  | 1 | |  |
| Ranunculaceae | *Caltha palustris* | Cal_pal | 2 | 2 | 3 | |  |  |  |  | 1 | 1 |  | 2 | 3 | |  |
| Ranunculaceae | *Ranuculus repens* | Ran_rep |  |  | 1 | |  |  |  |  |  |  |  |  | 1 | |  |
| Ranunculaceae | *Ranunculus acris* | Ran_sp |  |  |  | | 1 | 1 |  |  |  | 2 |  |  | 2 | |  |
| Ranunculaceae | *Ranunculus nivalis* | Ran_niv |  |  |  | | 1 |  | 1 |  |  |  |  |  | 1 | |  |
| Ranunculaceae | *Ranunculus subborealis* | Ran_sp |  |  |  | |  |  | 1 |  | 1 |  |  |  | 1 | |  |
| Ranunculaceae | *Thalictrum alpinum* | Tha_alp | 2 |  |  | | 2 |  | 1 |  | 1 | 1 | 1 | 1 | 2 | |  |
| Ranunculaceae | *Trollius europaeus* | Tro_eur |  |  | 2 | | 1 |  | 1 |  |  | 1 | 1 |  | 2 | |  |
| Rosaceae | *Alchemilla alpina* | Alc_alp |  |  |  | |  |  |  | 1 |  | 2 | 1 |  | 2 | |  |
| Rosaceae | *Alchemilla* cf. *glomerulans* | Alc_glo |  |  | 2 | |  |  | 1 |  |  | 2 |  |  | 2 | |  |
| Rosaceae | *Alchemilla* cf. *monticola* | ND |  |  |  | |  |  |  |  | 1 |  |  |  | 1 | |  |
| Rosaceae | *Alchemilla* cf. *wichurae* | ND |  | 1 |  | | 1 |  | 1 |  |  | 2 | 1 |  | 2 | |  |
| Rosaceae | *Alchemilla murbeckiana* | ND |  |  |  | |  |  |  | 1 |  |  | 1 |  | 1 | |  |
| Rosaceae | *Alchemilla* sp*.* | ID incomp |  | 2 | 2 | |  |  | 1 |  |  |  | 2 |  | 2 | |  |
| Rosaceae | *Comarum palustre* | Com_pal | 3 |  | 2 | | 1 | 3 |  | 2 | 2 | 1 | 1 | 3 | 3 | |  |
| Rosaceae | *Dryas octopetala* | Dry_oct |  | 2 |  | |  |  | 3 |  |  |  | 1 |  | 3 | |  |
| Rosaceae | *Filipendula ulmaria* | Fil_ulm | 2 |  | 2 | |  | 4 |  |  | 1 | 1 |  | 3 | 4 | |  |
| Rosaceae | *Geum rivale* | Geu_riv | 2 |  | 2 | |  | 2 |  |  |  | 1 |  |  | 2 | |  |
| Rosaceae | *Potentilla erecta* | Pote_ere |  |  | 2 | |  |  |  |  |  |  |  |  | 2 | |  |
| Rosaceae | *Potentilla* sp*.* | ID incomp |  |  |  | |  |  | 1 |  |  |  |  |  | 1 | |  |
| Rosaceae | *Rubus chamaemorus* | Rub_sp | 3 | 3 | 4 | | 2 | 3 |  | 2 | 3 | 2 | 2 | 3 | 4 | |  |
| Rosaceae | *Rubus idaeus* | Rub_ida |  |  |  | |  |  |  |  |  |  |  | 3 | 3 | |  |
| Rosaceae | *Rubus saxatilis* | Rub_sp | 2 | 2 |  | | 1 | 2 |  |  | 1 |  | 1 | 2 | 2 | |  |
| Rosaceae | *Sibbaldia procumbens* | Sib_pro |  |  |  | |  |  | 1 |  |  |  | 1 |  | 1 | |  |
| Rosaceae | *Sorbus aucuparia* | Sor_acu | 2 | 2 | 2 | | 1 | 1 |  | 1 | 1 | 3 |  | 3 | 3 | |  |
| Rubiaceae | *Galium* cf*. trifidum* | Gal_tri | 1 |  |  | |  |  |  |  |  |  |  |  | 1 | |  |
| Rubiaceae | *Galium palustre* | Gal_pal |  |  |  | |  |  |  |  |  |  |  | 1 | 1 | |  |
| Salicaceae | *Populus tremula* | Pop_tre | 2 | 3 |  | |  |  |  |  |  | 1 |  |  | 3 | |  |
| Salicaceae | *Salix arbuscula* | Salicac |  | 2 |  | |  |  |  |  |  |  |  | 2 | 2 | |  |
| Salicaceae | *Salix caprea* | Salicac |  |  |  | |  | 3 |  |  |  |  |  |  | 3 | |  |
| Salicaceae | *Salix* cf. *hastata* | Salicac |  |  |  | |  | 3 |  |  |  |  |  |  | 3 | |  |
| Salicaceae | *Salix glauca* | Salicac | 2 |  | 2 | | 2 | 3 | 3 | 2 | 1 | 2 | 4 |  | 4 | |  |
| Salicaceae | *Salix glauca* x *myrsinites* | Salicac |  |  |  | |  |  |  |  |  |  | 1 |  | 1 | |  |
| Salicaceae | *Salix herbacea* | Salicac |  |  |  | |  |  | 1 | 1 |  |  | 2 |  | 2 | |  |
| Salicaceae | *Salix herbacea* x *lanata* | Salicac |  |  |  | |  |  |  |  |  |  | 2 |  | 2 | |  |
| Salicaceae | *Salix lanata* | Salicac |  |  |  | | 1 |  | 3 |  |  |  | 4 |  | 4 | |  |
| Salicaceae | *Salix lapponum* | Salicac | 2 |  | 1 | | 1 | 3 |  | 2 | 1 | 1 | 3 | 2 | 3 | |  |
| Salicaceae | *Salix myrsinifolia* | Salicac | 2 |  | 1 | |  |  | 3 |  | 1 |  |  | 1 | 3 | |  |
| Salicaceae | *Salix myrsinites* | Salicac |  |  |  | |  |  |  |  | 1 |  |  |  | 1 | |  |
| Salicaceae | *Salix phylicifolia* | Salicac |  |  | 1 | | 1 |  |  |  | 1 |  | 2 |  | 2 | |  |
| Salicaceae | *Salix polaris* | Salicac |  |  |  | |  |  | 3 |  |  |  | 2 |  | 3 | |  |
| Salicaceae | *Salix reticulata* | Salicac |  | 2 |  | |  |  | 3 |  |  |  | 3 |  | 3 | |  |
| Salicaceae | *Salix* sp. total | Salicac | 1 | 3 | 2 | | 1 | 3 | 3 | 2 | 1 |  | 4 | 1 | 4 | |  |
| Saxifragaceae | *Micranthes hieracifolia* | Mic_hie |  |  |  | |  |  | 1 |  |  |  |  |  | 1 | |  |
| Saxifragaceae | *Micranthes stellaris* | Mic_ste |  |  |  | |  |  |  |  |  |  | 1 |  | 1 | |  |
| Saxifragaceae | *Saxifraga aizoides* | Sax_aiz |  | 3 |  | |  |  | 2 |  | 1 |  | 2 |  | 3 | |  |
| Saxifragaceae | *Saxifraga oppositifolia* | Sax_opp |  | 1 |  | |  |  | 1 |  |  |  |  |  | 1 | |  |
| Selaginellaceae | *Selaginella selaginoides* | ND | 2 | 2 |  | | 1 |  | 1 |  |  |  | 1 |  | 2 | |  |
| Sparganiaceae | *Sparganium angustifolium* | Spa_sp |  |  | 1 | |  |  |  | 3 | 4 | 3 |  |  | 4 | |  |
| Sparganiaceae | *Sparganium* *hyperboreum* | Spa_sp |  | 3 | 2 | | 3 |  |  | 1 |  |  |  | 2 | 3 | |  |
| Sparganiaceae | *Sparganium natans* | Spa_sp |  | 2 |  | |  |  |  |  |  |  |  |  | 2 | |  |
| Sparganiaceae | *Sparganium* sp. | Spa_sp |  |  |  | | 3 |  |  |  |  |  |  |  | 3 | |  |
| Thelypteridaceae | *Phegopteris connectilis* | Phe_con | 2 | 2 | 2 | | 1 | 2 |  | 2 | 1 | 1 |  | 2 | 2 | |  |
| Tofieldiaceae | *Tofieldia pusilla* | Tof_pus |  | 3 |  | | 1 | 1 | 1 |  | 1 | 1 | 1 |  | 3 | |  |
| Valerianaceae | *Valeriana sambucifolia* | Val_sam | 1 |  | 2 | |  |  |  |  |  | 1 |  | 2 | 2 | |  |
| Violaceae | *Viola* cf. *biflora* | Vio_bif | 2 |  |  | |  | 1 | 1 |  |  | 1 | 1 |  | 2 | |  |
| Violaceae | *Viola palustris* | Vio_sp |  |  | 2 | |  |  |  | 1 | 1 |  |  |  | 2 | |  |
| Violaceae | *Viola* sp. | ID incomp | 1 |  |  | | 1 |  |  |  |  |  |  | 1 | 1 | |  |
| Woodsiaceae | *Athyrium* cf*. distentifolium* | Ath_sp |  |  |  | |  |  |  | 1 |  |  |  |  | 1 | |  |
| Woodsiaceae | *Athyrium disentifolium* | Ath_sp |  |  |  | |  | 3 |  |  |  | 2 |  | 2 | 3 | |  |
| Woodsiaceae | *Athyrium filix-femina* | Ath_sp | 1 |  | 4 | | 2 | 3 |  |  | 1 | 2 | 1 |  | 4 | |  |
| Woodsiaceae | *Gymnocarpium dryopteris* | Gym_dry | 3 |  | 2 | | 1 | 2 |  | 2 | 1 | 2 |  | 3 | 3 | |  |
|  |  |  |  |  |  | |  |  |  |  |  |  |  |  |  | |  |
| Filtered out due to <100% match to reference library | | | | | | | | | | | | | | | |  | |
| Filtered out due to only found in samples taken below 2 cm sediment depth | | | | | | | | | | | | | | | |  | |
| Filtered out due to <10 reads or <3 PCR repeat in a lake sample and/or dut to higher average in negative control than sample | | | | | | | | | | | | | | | |  | |
| Identification in field incomplete | | | | | | | | | | | | | | | |  | |
| Laking in reference library | | | | | | | | | | | | | | | |  | |
| Neither in DNA nor vegetation records | | | | | | | | | | | | | | | |  | |
| Vegetation <2m and potentially >2m | | | | | | | | | | | | | | | |  | |
| Vegetation only > 2m | | | | | | | | | | | | | | | |  | |
| DNA and vegetation <2m | | | | | | | | | | | | | | | |  | |
| DNA and vegetation > 2m | | | | | | | | | | | | | | | |  | |
| DNA only | | | | | | | | | | | | | | | |  | |
