# Supplementary material for: Plant DNA metabarcoding of lake sediments: How does it represent the contemporary vegetation
Source: PLoS One. 2018 Apr 17;13(4):e0195403. doi: 10.1371/journal.pone.0195403 (PMC5903670; doi:10.1371/journal.pone.0195403)
Supplement: S2 Table — Six individually tagged PCR repeats were run for each sample, giving a total of 336 PCR samples. Numbers of sequences and unique sequences are given for applying the criteria to all sequences. (DOCX) [file pone.0195403.s003.docx]

**S2 Table. Number of sequence reads remaining after each filtering step** for 42 samples from modern lake sediment collected in northern Norway, 6 extraction negative controls, 6 PCR negative controls and 2 PCR positive controls. Six individually tagged PCR repeats were run for each sample, giving a total of 336 PCR samples. Numbers of sequences and unique sequences are given for applying the criteria to all sequences.

| Filtering steps | Program/command | Total seq. | Unique seq. | True positives lost |
| --- | --- | --- | --- | --- |
| Raw reads |  | 28 618 690 |  |  |
| Pairwise alignment | *illuminapairedend* | 26 226 777 |  |  |
| Assignment to samples | *ngsfilter* | 24 656 541 |  |  |
| Merged identical reads | *obiuniq* & *obiannotate* |  | 322 732 |  |
| Removal of reads with count =1 & <12 bp | *obigrep* |  |  |  |
| Identification & removal of PCR/sequencing errors | *obiclean* ratio 0.05 & *obigrep* | 20 876 037 | 27 715 |  |
| Keeping sequences with ≥ 98% match | *ecotag* & R | 12 706 536 | 581 |  |
| Keeping sequences with 100% match and overall >10 reads | R and Excel | 11 236 288 | 301 | Some records of *Arctostaphylos uva-ursi* and *Potamogeton* sp.*;* complete loss of *Orthilia secunda.* |
| Remove controls (PCR negatives and positive controls) from data | R | 6 523 638 | 279 |  |
| Excluding bryophytes | R | 6 417 680 | 257 |  |
| Excluding 2-8 cm samples | R | 2 592 750 | 209 | *Alchillea millefolium, Anthriscus sylvestris, Arctos alpinus, Avenella flexuosa, Chamerion angustifolium, Dactylorhiza* sp*., Linnea borealis, Luzula* sp*., Melampyrum pratense, Pedicularis lapponica, Poa annua,* Poaceae*, Ranunculus* sp*., Rhododendron lapponicum, Saussurea alpina, Trifolium repens* |
| > 10 reads; > 1 PCR repeat in a lake sample for a sequence; and mean number of reads in negative controls < mean number of reads in lakes | R | 2 503 262 | 59 | *Alchemilla glomerulans, Allium schoenoprasum, Angelica archangelica, A. sylvestris, Arctous alpinus, Arctostaphylos uva-ursi, Astragalus alpinus, Bartsia alpine, Bistorta vivipara, Callitriche palustris, Carex paupercula, C. vaginata, Cirsium heterophyllum, Diapensia lapponica, Elymus caninus, Eriophorum angustifolium, Euphrasia* sp.*, Geum rivale, Melampyrum sylvaticum, Nardus stricta, Parnassia palustris, Picea abies, Pinus sylvestris, Potentilla erecta, Pyrola minor, P. rotundifolia, Ranunculus repens, Rumex* sp., *Saussurea alpina, Solidago virgaurea, Thalictrum alpinum, Trientalis europaea, Trollius europaeus, Valeriana sambucifolia, Veronica alpina, Viola biflora* |
| Remove taxa not in the region | R | 2 500 138 | 56 |  |
