# Supplementary material for: Plant DNA metabarcoding of lake sediments: How does it represent the contemporary vegetation
Source: PLoS One. 2018 Apr 17;13(4):e0195403. doi: 10.1371/journal.pone.0195403 (PMC5903670; doi:10.1371/journal.pone.0195403)
Supplement: S5 Table — The file consisted of 12706536 reads of 581 sequences, 98% match). Note that not all taxa used in the positive controls were present in the reference library but they match to closely related taxa. (DOCX) [file pone.0195403.s006.docx]

**S5 Table.** **Retrieval of positive controls from raw Orbitool output file.** The file consisted of 12706536 reads of 581 sequences, 98% match). Note that not all taxa used in the positive controls were present in the reference library but they match to closely related taxa.

| Family | Species | DNA conc. (ng/µL) | DNA taxa comment | DNA reads | DNA repeats |
| --- | --- | --- | --- | --- | --- |
| Asteraceae | *Leptinella plumosa* | 22.25 | Asteraceae (r117), *Leptinella squelida* + more taxa (blast) | 451726 | 12 |
| Caryophyllaceae | *Sagina procumbens* | 7.42 | *Sagina* (r117) | 458 334 | 12 |
| Poaceae | *Holcus lanatus* | 2.47 | *Holcus* (r117) | 66 080 | 12 |
| Caryophyllaceae | *Colobanthus kerguelensis* | 0.82 | 4 clusters of Caryophyllaceae found in all 12 repeats (r117) \| *Colobanthus quitensis* (blast). | 1568 | 12 |
| Plantaginaceae | *Callitriche antarctica* | 0.27 | *Callitriche hermaphroditica* (r117) | 15669 | 12 |
| Rosaceae | *Acaena magellanica* | 0.09 | Sanguisorbinae (r117) \| *Acaena* spp. (blast) | 17 776 | 12 |
| Brassicaceae | *Pringlea antiscorbutica* | 0.03 | Brassicaceae (r117) *Pringlea antiscorbutica* + other taxa (blast) | 5 271 | 12 |
| Poaceae | *Aira praecox* | 0.01 | A few Poaceae clusters but no match against the genebank reference sequence for *Aira* | 0 | 0 |
