# Supplementary material for: Plant DNA metabarcoding of lake sediments: How does it represent the contemporary vegetation
Source: PLoS One. 2018 Apr 17;13(4):e0195403. doi: 10.1371/journal.pone.0195403 (PMC5903670; doi:10.1371/journal.pone.0195403)
Supplement: S6 Table — The probability that all taxa in the vegetation were recorded (Vegetation), and that the DNA records represents true and false positives. Mean probability, standard deviation (SD) are given for each lake. (DOCX) [file pone.0195403.s007.docx]

**S6 Table. The probability of detection in eDNA and vegetation.** The probability that all taxa in the vegetation were recorded (Vegetation), and that the DNA records represents true and false positives. Mean probability, standard deviation (SD) are given for each lake.

| Lake |  | Vegetation detectability | |  | DNA false positive | | | DNA true positive | | |
| --- | --- | --- | --- | --- | --- | --- | --- | --- | --- | --- |
|  | Mean | | SD |  | Mean | SD |  | | Mean | SD |
| A-tjern | 0.933 | | 0.050 |  | 0.232 | 0.102 |  | | 0.898 | 0.056 |
| Brennskogtjønna | 0.921 | | 0.054 |  | 0.262 | 0.100 |  | | 0.668 | 0.089 |
| Einletvatnet | 0.927 | | 0.052 |  | 0.221 | 0.099 |  | | 0.823 | 0.071 |
| Finnvatnet | 0.879 | | 0.054 |  | 0.187 | 0.084 |  | | 0.550 | 0.109 |
| Gauptjern | 0.909 | | 0.055 |  | 0.187 | 0.091 |  | | 0.721 | 0.088 |
| Jula Jávri | 0.853 | | 0.045 |  | 0.205 | 0.081 |  | | 0.332 | 0.118 |
| Lakselvhøgda | 0.910 | | 0.055 |  | 0.059 | 0.049 |  | | 0.422 | 0.101 |
| Lauvås | 0.903 | | 0.056 |  | 0.211 | 0.087 |  | | 0.332 | 0.095 |
| Øvre Æråsvatnet | 0.946 | | 0.044 |  | 0.242 | 0.105 |  | | 0.656 | 0.083 |
| Paulan Jávri | 0.862 | | 0.049 |  | 0.179 | 0.084 |  | | 0.647 | 0.113 |
| Rottjern | 0.916 | | 0.055 |  | 0.332 | 0.105 |  | | 0.693 | 0.089 |
|  |  |  |  |  |  |  |  |  |  |  |
